# Supplementary material for: Prevalence and risk factors for antimicrobial resistance among newborns with gram-negative sepsis
Source: PLoS One. 2021 Aug 3;16(8):e0255410. doi: 10.1371/journal.pone.0255410 (PMC8330902; doi:10.1371/journal.pone.0255410)
Supplement: Attachment — Submitted filename: Response to reviewers 31May2021.docx [file pone.0255410.s002.docx]

**Response to reviewers**

Thank you for taking the time to comment on this manuscript. Your comments are highly constructive and appreciated.

Reviewer #1

1. How frequently does the study hospital change its antibiotic policy?

Response: Thank you, changes to antibiotic policy in the study hospital are made based on recommendations and policy changes from Ministry of Health and does not occur regularly, the last change to antibiotic policy occurred in 2014.

2. The effective sample size was 119. Total number of newborn enrolled i.e. 4828 was not used for analysis. Therefore, it may not be included in abstract.

Response: Thank you, this was removed in the abstract.

Reviewer #2

1. Please see the attached document with my comments, suggestions, and questions. Please consider these and answer the questions as best you can

Response: Thank you for comments and suggestions. We have addressed them as best we can. Following recommendations from CDC website and Cochrane, "Gram should be capitalized and never hyphenated when used as Gram stain; gram negative and gram positive should be lowercase and only hyphenated when used as a unit modifier", we did not capitalize the “g” in gram-negative but accepted all other suggested edits.

2. A note about the figures and tables:

The authors are encouraged to make a list all figures at the end of the document. Moreover, include figure captions, or legends, that are complete so that the reader is able to understand the figure without reference to the text. Describe only what is seen on the image. Use of arrows, asterisks, arrowheads, etc. are useful to communicate or covey to the readers your findings. The authors are advised to revise the captions all figures.

Response: The figure legends and captions have now been revised as advised. The figures are cited in the text in order per the PLOSone formatting instructions and uploaded as sperate individual figure files. <https://journals.plos.org/plosone/s/file?id=wjVg/PLOSOne_formatting_sample_main_body.pdf>, we have added a list of all figures to the end of the document as suggested. We understand colors are typically part of the legend, however we kept the legends defining the colors used in the figure in the figures in case this is easier to format in the publication. Please let us know if you’d prefer that we move these into the manuscript.

The authors are encouraged to make a list all tables at the end of the document. The headings or titles of the tables should be complete so that the reader is able to understand the table without reference to the text. The authors are advised to revise the titles of all tables. For example: “Table 1.Prevalence and risk factors for antimicrobial resistance among newborns with Gram-negative sepsis: Descriptive analysis for baseline characteristics of 119 newborns Included in Antimicrobial Resistance Study”

Response: The table titles and legends have now been revised as advised. We have added a list of tables to the end of the document as per the reviewer’s comment.

3. Why 6 drops and not 5 or 7? What is the reasoning? Please explain.

During protocol development 6 drops was agreed as a guide to allow sufficient volume of blood to adhere to the agar and encourage microbial growth. It might be best to use ‘aliquot’ rather than being too detailed, hence the wording has been changed accordingly.

Thank you your time and helpful comments to strengthen the manuscript.
