# Supplementary material for: Prevalence and risk factors for antimicrobial resistance among newborns with gram-negative sepsis
Source: PLoS One. 2021 Aug 3;16(8):e0255410. doi: 10.1371/journal.pone.0255410 (PMC8330902; doi:10.1371/journal.pone.0255410)
Supplement: Attachment — Submitted filename: BARNARDS AMR Manuscript 28Feb_with Reviewer comments and edits.docx [file pone.0255410.s001.docx]

**Prevalence and risk factors for antimicrobial resistance among newborns with Gram-negative sepsis**

Semaria Solomon^1‡^, Oluwasefunmi Akeju^2‡^, Oludare A. Odumade^3^, Rozina Ambachew^1^, Zenebe Gebreyohannes^1^, Kimi Van Wickle^2^, Mahlet Abayneh^1^, Gesit Metaferia^1^, Maria J Carvalho^4,5^, Kathryn Thomson^4^, Kirsty Sands^4,6^, Timothy R Walsh^4,7^, Rebecca Milton^4,8^, Frederick G B Goddard^2^, Delayehu Bekele^1,2^, Grace J Chan^1,2,3^

^1^St. Paul’s Hospital Millennium Medical College, Ethiopia

^2^Harvard T.H. Chan School of Public Health, Boston, MA, USA

^3^Boston Children’s Hospital, Harvard Medical School, Boston, MA, USA

^4^Division of Infection and Immunity, Cardiff University, UK

^5^Institute of Biomedicine, Department of Medical Sciences, University of Aveiro, Portugal

^6^Department of Zoology, University of Oxford

^7^Ineos Oxford Institute of Antimicrobial Research, Department of Zoology, University of Oxford

^8^Centre for Trials Research, Cardiff University, Cardiff

‡ Equally contributed to this work

*Correspondence to:

Grace J Chan, MD PhD

Department of Epidemiology

Harvard T.H. Chan School of Public Health

Boston, MA 02115, USA

**Abstract**

**Introduction**

Newborn sepsis accounts for more than a third of neonatal deaths globally and one in five neonatal deaths in Ethiopia. The first-line treatment recommended by WHO is the combination of gentamicin with ampicillin or benzylpenicillin. Gram-negative bacteria (GNB) are increasingly resistant to previously effective antibiotics.

**Objectives**

Our goal was to estimate the prevalence of antibiotic-resistant Gram-negative bacteremia and identify risk factors for antibiotic resistance, among newborns with GNB sepsis.

**Methods**

At a tertiary hospital in Ethiopia, we enrolled a cohort of 4,589 mothers and their 4,828 newborns, between March and December 2017, who were followed up until 60 days of life. Among the newborns with clinical signs of sepsis, blood samples were cultured; bacterial species were identified and tested for antibiotic susceptibility. We described the prevalence of antibiotic resistance, identified newborn, maternal and environmental factors associated with multidrug resistance (MDR), and combined resistance to ampicillin and gentamicin (AmpGen), using multivariable regression.

**Results**

Of the 119 newborns with Gram-negative bacteremia, 80 (67%) were born preterm and 82 (70%) had early-onset sepsis. The most prevalent gram-negative species were *Klebsiella pneumoniae* 94 (79%) followed by *Escherichia coli* 10 (8%). Ampicillin resistance was found in 113 cases (95%), cefotaxime 104 (87%), gentamicin 101 (85%), AmpGen 101 (85%), piperacillin-tazobactam 47 (39%), amikacin 10 (8.4%) and Imipenem 1 (0.8%). Prevalence of MDR was 88% (n=105). Low birthweight and late-onset sepsis (LOS) were associated with higher risks of AmpGen-resistant infections. All-cause mortality was higher among newborns treated with ineffective antibiotics.

**Conclusion**

There was significant resistance to current first-line antibiotics and cephalosporins. Additional data are needed from primary care and community settings. Amikacin and piperacillin-tazobactam had lower rates of resistance; however, context-specific assessments of their potential adverse effects, their local availability, and cost-effectiveness would be necessary before selecting a new first-line regimen to help guide clinical decision-making.

**Introduction**

More than 2.4 million neonatal deaths occur each year globally, the majority of which occur in low and middle-income countries (LMIC)[1]. About one-third of newborn deaths are caused by systemic infections, also referred to as neonatal sepsis[2, 3]. Ethiopia currently has a high neonatal mortality rate, which in 2018 was estimated at 28 per 1,000 live births and newborn sepsis accounts for about one in five neonatal deaths[4, 5]. Gram-negative bacteria (GNB) are estimated to be responsible for up to two-thirds of neonatal sepsis in Ethiopia [6-10]. Previous research suggests that the most predominant GNB isolates found among newborns with sepsis are *Klebsiella pneumoniae* and *Escherichia coli (E. coli)*[7, 9]. GNB are becoming increasingly resistant to previously effective antibiotics as new resistance genes can be readily transferred across GNB by mobile genetic elements[11]. An example is the plasmid-mediated inter-genus transfer of the resistance genes for extended spectrum beta-lactamases, which has been observed between *Escherichia coli* and *Klebsiella pneumoniae* likewise among other organisms in the *Enterobacteriaceae* family [12]. As a result, neonatal sepsis caused by GNB is twice as fatal as neonatal sepsis arising from gram-positive bacteria (GPB)[13-16]. The first line antibiotic therapy recommended by World Health Organization (WHO) to treat neonatal sepsis is a combination of intravenous or intramuscular gentamicin and benzylpenicillin or ampicillin, which is also the most common treatment of sepsis in infants under two months old in Ethiopia, [17, 18]. Third-generation cephalosporins, such as cefotaxime, ceftriaxone, and ceftazidime, are frequently used alternatives when resistance to first-line antibiotics is suspected[19].

Inappropriate antibiotic treatment for sepsis, due to antimicrobial resistance (AMR), has been linked to increased neonatal mortality and up to 30% of deaths from neonatal sepsis have been attributed to AMR[15, 20]. There have been reports of increasing rates of resistance to first-line and alternative therapies in some sub-Saharan African (sSA) countries[21-24]. A study from Ethiopia reported 91% of *Klebsiella spp.* and 67% of *E. coli* were resistant to ampicillin, and 82% and 56% resistant to gentamicin, respectively[7]. Furthermore, two studies reported high rates of resistance to third-generation cephalosporins; however, both findings came from observing very few GNB isolates (n=24, n=14)[7, 9]. Studies have reported multi-drug resistance (MDR), defined as acquired resistance to at least one agent from three or more categories of antibiotics, to be greater than 70% for GNB[7, 9, 10, 25, 26]. However, there is a paucity of data from Ethiopia on the sensitivity of organisms to carbapenems and other infrequently used antibiotics.

There are known risk factors for antimicrobial resistance. Preterm birth, prolonged rupture of membranes, maternal infections, and prolonged hospitalization are some of the previously identified risk factors for neonatal sepsis[27-30]. Frequent antibiotic use, poor sanitation and hygiene, and poor compliance with infection control practices have been associated with an increased incidence of AMR[31-33]. Host factors could specifically predispose a newborn to infections by antibiotic-resistant GNB. Several studies have reported an increased risk of neonatal infections by antibiotic-resistant pathogens with intrapartum exposure to ampicillin[34-38]. Penicillins are typically the recommended intrapartum antibiotic given to women with group B Streptococci (GBS) colonization to reduce the risk of early neonatal sepsis[39]. Currently, there are limited data on the association between the incidence of antibiotic-resistant neonatal infections and other maternally administered antibiotics during labor. Understanding this relationship may guide the selection of intrapartum antibiotics.

The objective of this study was to estimate the prevalence of single and multidrug phenotypic resistance to 19 antibiotics, among GNB isolates from newborns with sepsis in Ethiopia. This data along with the subsequent follow-up of newborns from birth through 60 days after birth were used to identify neonatal, maternal, and environmental risk factors for AMR among newborns with gram-negative sepsis. This study provides evidence to inform future decisions and recommendations for treatment of newborn sepsis in Ethiopia and LMIC countries.

**Methods**

*Study design and study population*

As part of a multi-country study, Burden of Antibiotic Resistance in Newborns from Developing Societies (BARNARDS), we enrolled a cohort of mothers and their newborns at time of birth and followed them through 60 days of life between March 2017 and December 2017 at St. Paul’s Hospital Millennium Medical College (SPHMMC), Addis Ababa Ethiopia. We included newborns delivered at SPHMMC (inborn) and those born elsewhere and received care at SPHMMC (outborn). Among the newborns with clinical signs of sepsis, we obtained blood cultures to test for bacterial and fungal growth. In this secondary data analysis of AMR, we included a cohort of newborns with laboratory confirmed gram-negative sepsis. We excluded newborns without laboratory-confirmed sepsis, newborns whose blood cultures did not yield GNB isolates, and newborns who had no results for antibiotic susceptibility testing on their isolates (**Fig 1**).

**Fig 1.** Diagram showing the enrollment cascade for the study cohort

*Ethical approval*

Mothers enrolled in the BARNARDS-Ethiopia study provided informed consent for themselves and their newborns. The study protocol was reviewed and approved by the ethical review committees of St. Paul’s Hospital Millennium Medical College, Boston Children’s Hospital, Harvard T.H. Chan School of Public Health.

*Exposures and outcomes*

The primary outcome of interest was the prevalence of resistant phenotypes of GNB isolates to 19 antibiotics, distinct antibiotic combinations, and multiple antibiotics. The nine classes of antibiotics assessed were penicillins (ampicillin, amoxicillin/clavulanic acid, piperacillin/tazobactam); cephalosporins (cefotaxime, ceftriaxone, ceftazidime, cefepime); carbapenems (meropenem, imipenem, and ertapenem); monobactams (aztreonam); aminoglycosides (gentamicin, amikacin, and tobramycin); fluoroquinolones (ciprofloxacin and levofloxacin); tetracyclines (tigecycline); fosfomycin; and polymyxin (colistin). An isolate was considered to be resistant to a class of antibiotics when it was resistant to at least one antimicrobial agent within that class[26]. Based on these nine classes of antibiotics, we created an MDR score that represented the number of antibiotic classes considered in our study that a GNB isolate is resistant to, on a scale of 0-9.

We considered ampicillin and gentamicin, ampicillin and cefotaxime, and piperacillin-tazobactam and amikacin as combinations, based on the high efficacy reported in a previous study[19, 40]. Resistance to an antibiotic combination was assumed when an isolate demonstrated resistance *in vitro* to each of the antibiotics that constitute the combination. We compared the 28-day and 60-day all-cause mortality rates between newborns who had been treated with antibiotic combinations to which the pathogen was later determined to be resistant and newborns who received antibiotics to which their infection was susceptible.

To assess the association between exposure to intrapartum antibiotics and AMR in neonatal sepsis, we defined the exposure as any antibiotic administered to mothers during labor and delivery. The outcome was the prevalence of single-drug resistance to the intrapartum antibiotic among newborns with gram-negative sepsis.

In the analysis of the AMR risk factors, the exposures were categorized as:

1) Neonatal characteristics: birth cohort (inborn, outborn); sex (male, female); birthweight as binary (Normal ≥2500g; Low <2500g) and categorical (normal ≥2500g, low 1500g to <2500g, very low <1500g); gestational age as binary (term ≥37 weeks, preterm <37 weeks), and categorical (term ≥37 weeks, moderate to late preterm 32 to <37 weeks, very preterm <32 weeks); and type of delivery (vaginal or caesarean).
2) Onset of clinical sepsis (early-onset sepsis or EOS <72 hours of life, late-onset sepsis or LOS ≥72 hours to 28 days).
3) Factors relating to maternal antibiotic usage: prenatal antibiotic use during the last three months of pregnancy (yes, no); intrapartum antibiotic use (yes, no).
4) Sanitation and Hygiene factors: type of toilet in the home (standard flush toilet, squat toilet/pit latrine, communal toilet outside the home/others); and access to running water (less than once/week, irregular or 2-3times/week, ≥4-6times/week).

The outcomes were resistance to both ampicillin and gentamicin (AmpGen) and MDR score (0-9). **Fig 2** highlights the conceptual framework showing the associations between exposure and outcome variables.

**Fig 2**. Conceptual framework showing associations between exposure and outcome variables

*Data collection*

Research staff collected standard of care data on maternal and neonatal clinical parameters from clinical records, interviews and direct observation of mothers and newborns. Study data collectors conducted home visits on the 3^rd^, 7^th^, and 28^th^ days of life to examine newborns for symptoms of neonatal sepsis and phone visit on day 60 for maternal report of symptoms and vital status outcomes. Neonatal blood samples for cultures were collected, stored, and analyzed according to best practices.

*Laboratory analysis*

Upon clinical signs of sepsis, a neonatal blood sample was collected and subjected to analysis using an automated blood culture system (BACTEC™, BD). Following a positive blood culture indication, six drops of blood were transferred to Columbia Blood Agar supplemented with 5% sterile blood and incubated at 37°C overnight. Preliminary identification at SPHMMC was performed using Gram-staining and Enterosystem 18R (for Enterobacterales). All isolates were stored on charcoal swabs and kept at 2-8°C for shipment to the Cardiff University, UK, as part of BARNARDS, and in compliance with UN3373 regulations. Thereafter, species identification and genomic characterization was performed using whole-genome sequencing and agar dilution was carried out to determine minimum inhibitory concentrations (MIC), the lowest concentration of an antibiotic that inhibits bacteria’s growth. Phenotypes of isolates were classified according to the breakpoints for MIC, as recommended by the European Committee on Antimicrobial Susceptibility Testing (EUCAST)[41], as part of BARNARDS (*Sands et al.*, in press). An isolate’s phenotype was categorized as "S-Susceptible using standard dosing regimen” when there was a high likelihood of therapeutic success using a standard dosing regimen of the agent; “I-Susceptible with increased exposure” when there was a high likelihood of therapeutic success because exposure to the agent is increased by adjusting the dosing regimen or by its concentration at the site of infection; "R-Resistant" when there is a high likelihood of therapeutic failure even when there is increased exposure[41].

*Statistical analysis*

We used descriptive statistics to summarize exposure variables. We estimated the prevalence of GNB isolates resistant to each of 19 antibiotics tested and selected antibiotic combinations and the prevalence of MDR. Using a one-sided test of proportion, we assessed whether treating neonatal sepsis with antibiotic combinations that the causative GNB were resistant to, was associated with increased all-cause mortality at 28 and 60 days of life. We used a one-sided test of proportion to examine whether the prevalence of resistant phenotypes to each intrapartum antibiotic used in this study was higher among newborns with a history of maternal exposure to the specific antibiotics.

Since gram-negative bacteria are associated with an increased risk of AMR (**Fig 2**), we conducted our study within the subgroup of newborns with GNB sepsis when identifying risk factors for AMR sepsis. We used multivariable regression models to identify and adjust for possible sources of potential confounding among exposure variables.

We assessed the relationship between exposure variables and AmpGen using univariable and multivariable log-binomial regressions. We assessed the association between exposure variables and MDR score using univariable and multivariable linear regressions. We selected covariates for the multivariable models using the purposeful selection method[42]. We included candidate covariates with p-values <0.25 from the univariable analysis in a multivariable model and those with a p-value of >0.1 were dropped if their exclusion did not result in >20% change in the effect estimate of other covariates. We added covariates with p-value >0.25 in the univariable analysis, if they had p-values <0.1 in the multivariable model. We excluded covariates with ≥20% observations missing from all analyses. We analyzed all data using STATA 16.1 and set statistical significance at a p-value of <0.05.

**Results**

*Summary characteristics*

Between March 2017 and December 2017, as part of BARNARDS-Ethiopia, 4,589 mothers and their 4,828 newborns (inborn=4,583, outborn=705) were enrolled and samples were obtained for blood cultures from 1,020 newborns (inborn=479; outborn=541) with clinical signs of sepsis. There were 443 newborns (inborn=183; outborn=260) with positive blood cultures. Of these, 300 isolates retrieved from samples of 289 newborns revealed the prevalence of GNB sepsis as 50% (n=150), GPB 47% (n=141) and fungal infections 3% (n=9). Of the 11 newborns with more than one pathogen, four had a GNB and an unspecified GPB; one had both GPB and fungus while the additional pair of isolates or species in the remaining were duplicates of the original. Whole-genome sequencing was used to identify 121 GNB species from isolates belonging to 121 newborns. Two newborns were excluded because of insufficient data on AST (**Fig 1**). Our cohort for the AMR study consisted of 119 newborns, 70 (59%) outborn and 49 (41%) inborn.

Of the 119 newborns, 53 (45%) were females, 53 (45%) were males, and sex was unreported for 13 (11%) newborns (**Table 1**). The majority of newborns (n=80; 67%) were born preterm, of which 77 (96%) of the preterm newborns had low birthweight. Of the term newborns, 15 out of 39 (38%) were low birthweight. Early-onset sepsis (EOS) was identified in 56 out of 80 (70%) preterm and 26 out of 39 (67%) term newborns. For all newborns, the median time to clinical sepsis from delivery was one day (IQR =0, 4 days). Nearly all (n=118; 99%) newborns with GNB sepsis were hospitalized and blood samples had been collected from most of them (n=99; 84%) for suspected sepsis prior to or on the day of hospitalization. The most predominant GNB species identified were *Klebsiella pneumoniae* (*Kp*) (n=94; 79%)*,* followed by *Escherichia coli* (n=10; 8%), and *Acinetobacter baumannii* (n=6; 5%) (**Fig 3**). There were 11 sequence types (ST) and capsular-antigen serotypes (KL) of *Kp* identified, the most predominant being ST35/KL108 (n=38), ST37/KL15 (n=28), ST218/KL57 (n=10) and ST985/KL39 (n=8), which were responsible for 89% (n=84/94) of all *Kp* infections (**Fig 4**). Incidence of ST35 during the study period was similar for inborn and outborn while the incidence of ST37 was twice among inborn (33%) compared to outborn (17%) and eight of the ten newborns who had ST218 were outborn. Newborns delivered vaginally had higher incidence of ST35 and ST218 while increased incidence of ST37 was observed with Caesarean delivery (**Fig 5**). New infections by other *Kp* strains and non-*Kp* GNB were either sporadic (when rare) or fairly distributed over the entire study period, while ST35, ST37, ST218 and ST985 were more clustered, with at least 70% of cases occurring within two months(**Fig 6**).

**Table 1**: Descriptive analysis for baseline characteristics of 119 newborns Included in AMR Study

| Characteristics | N=119 | Percentages (%) |
| --- | --- | --- |
| Median age at diagnosis in days for all newborns (IQR)  Median age at diagnosis for newborns with EOS (IQR)  Median age at diagnosis for newborns with LOS (IQR) | 1 (0, 4)  0 (0, 1)  6 (4, 6) |  |
| Sex  Female  Male  Missing | 53  53  13 | 45  45  11 |
| Newborn Term  Term (37-42weeks)  Preterm (<37weeks)  Moderate/Late Preterm (32-<37 weeks)  Very/Extremely Preterm (<32weeks) | 39  80  62  18 | 33  67  52  15 |
| Birthweight  Normal birthweight (≥2500g)  Low birthweight (1500-<2500g)  Very low/Extremely low birthweight (<1500g) | 27  52  40 | 23  44  34 |
| Birth Cohort  Inborn  Outborn | 49  70 | 41  59 |
| Type of Delivery  Vaginal  Caesarian Section  Missing | 60  34  25 | 50  29  21 |
| Type of Sepsis  Early Onset  Late Onset  Missing | 82  35  2 | 69  29  2 |
| Antibiotics Administered to Newborns  Ampicillin & Gentamicin  Ampicillin & Cefotaxime  Ampicillin & Ceftriaxone  Vancomycin & Ceftazidime  No antibiotic received  Missing | 83  11  1  5  12  7 | 70  9  1  4  10  6 |
| Intrapartum Antibiotics  No  Yes  Missing | 72  25  22 | 61  21  18 |
| Prenatal Antibiotics  No  Yes  Missing | 101  4  14 | 85  3  12 |

**Fig 3**. Prevalence of gram-negative bacterial species

**Fig 4.** Distribution of *Klebsiella spp* (n=100) and the clonal strains of *Klebsiella pneumoniae* (n=94)

**Fig 5**. Distribution of the clonal strains of *Klebsiella pneumoniae* by newborns subgroups

**Fig 6.** Incidence of *Klebsiella pneumoniae* strains over the study period (Mar. 2017 to Feb. 2018)

*Prevalence of AMR*

Among the penicillin class of antibiotics, ampicillin-resistance was observed in 113 (95%) isolates while resistance to piperacillin-tazobactam was found in 47 (39%) isolates (**Fig 7**). For aminoglycosides, resistance was high to gentamicin (n=101; 85%) but very low to amikacin (n=1; <1%). There was a high prevalence of resistance to all third and fourth-generation cephalosporins tested. Resistance to ceftriaxone was found in 105 (88%) isolates, cefotaxime 104 (87%), ceftazidime 104 (87%) and cefepime 101 (85%). Resistance to ciprofloxacin, a fluoroquinolone, was observed in 50 (42%) isolates. Nearly all isolates were susceptible to imipenem (n=118; 99%) and meropenem (n=118; 99%). All isolates were susceptible to colistin. Most isolates were resistant to AmpGen (n=101; 85%), the recommended first-line therapy, likewise ampicillin and cefotaxime (n=104; 87%), another antibiotic combination used for treating sepsis among study participants. Among newborns with EOS, AmpGen-resistance was higher among preterm (91%) than term (65%) newborns, and this finding was similar for MDR (**Fig 8**). Prevalence of MDR was 88% (n=105) and the median number of antibiotic classes in which an isolate demonstrated resistance to at least one drug, was 4 (IQR=4, 5).

**Fig 7.** Prevalence of resistant phenotypes of gram-negative isolates

**Fig 8.** Prevalence of MDR and AmpGen resistance among newborns with GNB sepsis

*Antimicrobial therapy and treatment outcomes*

Of the 119 newborns with blood culture confirmed sepsis, 100 (84%) received antibiotics. Antibiotic data were missing for seven newborns and 12 did not receive antibiotics due to death occurring shortly after admission, parental refusal of antibiotics and discharge from hospital against medical advice. Ampicillin was administered to 95 out of 100 newborns who received antibiotics (**Fig 9**). Among the 83 newborns who were administered ampicillin and gentamicin, 72 (87%) had AmpGen-resistant infections. Eight out of the 11 newborns treated with ampicillin and cefotaxime had GNB phenotypes resistant to this antibiotic combination. Of 119 newborns with GNB sepsis, 30 (25%) died by their 28th day of life. At day 60, 72 (61%) were alive, 43 (36%) dead and 4 (3%) were lost to follow-up (**Fig 10, Fig 11**). Compared to AmpGen-susceptible infections, AmpGen-resistant infections were associated with higher 28-day (n=2/18, 11%; versus n=28/97, 29%; p=0.058) and 60-day (n=4/18, 22%; versus n=39/97, 40%; p=0.074) all-cause mortality, although the differences observed were not statistically significant. The prevalence of 28-day and 60-day all-cause mortality was significantly higher among newborns who had received antibiotics to which their GNB infections were resistant (29% and 41%, respectively) compared to those whose treatment matched their infections (0% and 14%, respectively) (p=0.010, p=0.028). These findings were consistent among preterm and term newborns, inborn and outborn, newborns with EOS or LOS, regardless of the antibiotic combinations used. 28-day all-cause mortality among newborns who were treated with AmpGen for AmpGen-resistant infections (29%) was similar to that observed among newborns without evidence of receiving antibiotics (31%, p=0.839). Nearly all the *Kp* strains (97%) were resistant to AmpGen, but the 60-day all-cause mortality among newborns infected with *Kp* strains were not much different from the average in this study except for ST218 where eight out of the 10 infected newborns died (**Fig 12**).

**Fig 9**. Proportion of newborns with GNB infections resistant to the antibiotics they received

**Fig 10**. 28-day mortality among term and preterm newborns who received antibiotics for which their GNB infections were resistant

**Fig 11**. 60-Day cumulative outcomes for all newborns with GNB sepsis based on the antibiotics received

**Fig 12**. 60-day cumulative outcomes for all newborns by species and strains of GNB

*Risk factors for AMR among all newborns*

Of the 97 newborns with complete data, 25 (26%) were exposed to intrapartum antibiotics (Ampicillin, n=11; and Ceftriaxone, n=14). Intrapartum exposure to ampicillin (n=11) and ceftriaxone (n=14) were associated with a higher prevalence of infections with ampicillin-resistant (100% vs. 94%, p=0.20) and ceftriaxone-resistant (93% vs. 86%, p=0.24) GNB phenotypes, respectively (**Fig 13**). However, neither of these associations was statistically significant. The prevalence of prenatal antibiotic use was low (n=4/119; 3%) in this study hence, its possible relationship with AMR was not assessed.

**Fig 13**. Associations between exposure to intrapartum antibiotics and single-drug resistance

Compared to term newborns, preterm newborns had a 27% greater risk of infections with GNB that were resistant to AmpGen, (RR= 1.27; 95% CI= 1.03, 1.56) (**Table 2**). The risk of AmpGen resistance was 1.15 times (95% CI= 0.96, 1.39) higher among newborns from households whose access to running water was less than one day per week compared to newborns whose households had running water at least four days per week. The outborn did not have a significantly lower risk of AmpGen resistance compared to the inborn (RR=0.94; 95% CI= 0.81, 1.10). The multivariable model for AmpGen resistance consisted of sex, LBW, and LOS. Preterm birth was excluded as it was found to be collinear with birthweight. After adjusting for low birthweight and LOS, the risk of AmpGen-resistant infections was 15% (RR= 0.85; 95% CI= 0.76, 0.96) lower among female newborns as compared to male newborns. Low birthweight and LOS were associated with 36% (RR= 1.36; 95% CI= 1.02, 1.83) and 13% (RR= 1.13; 95% CI= 1.03, 1.23) higher risks of AmpGen-resistant infections, respectively.

**Table 2**: Associations between neonatal, maternal and environmental factors and combined resistance to ampicillin and gentamicin (AmpGen) among 119 newborns with GNB sepsis

|  | Univariable Analysis | | | | Multivariable Model | | | | | | | |
| --- | --- | --- | --- | --- | --- | --- | --- | --- | --- | --- | --- | --- |
| Variable | **Risk Ratio** | | **95% CI** | **p-value** | **Variable** | **Risk Ratio** | | **95% CI** | | **p-value** | | |
| Newborns’ Characteristics |  |  | |  |  |  | |  | |  | | |
| Female (ref: male) | 0.91 | | 0.78, 1.08 | 0.281 | Female | 0.85 | | 0.76, 0.96 | | 0.010 | | |
| Preterm (binary)  <37 weeks (ref: ≥37weeks)  Preterm (categorical)  (ref: ≥37weeks)  Moderate/Late (32-<37  weeks)  Very/Extremely (<32weeks) | 1.27  1.28  1.24 | | 1.03, 1.56  1.04, 1.58  0.96, 1.60 | 0.024  0.021  0.102 |  |  | |  | |  | | |
| Low Birthweight (binary)  <2500g (ref: normal ≥2500g)  Birthweight (categorical)  (ref: normal ≥2500g)  Low (1500-<2500g)  Very Low/Extremely Low  (<1500g) | 1.35  1.36  1.35 | | 1.03, 1.78  1.02, 1.80  1.01, 1.80 | 0.031  0.034  0.040 | Low birthweight as binary (<2500g) | 1.36 | | | 1.02, 1.83 | | 0.039 | |
| Outborn (ref: inborn) | 0.94 | | 0.81, 1.10 | 0.451 |  | | | | | | | |
| Caesarian delivery (ref: vaginal) | 0.97 | | 0.82, 1.14 | 0.681 |  |  |  |  |  |  |  |  |
| Onset of Sepsis |  | |  |  |  | | | | | | | |
| Late-onset sepsis (ref: early-onset sepsis) | 1.10 | | 0.96, 1.27 | 0.175 | Late-onset sepsis | | 1.13 | | 1.03, 1.23 | | | 0.007 |
| Maternal Antibiotics |  | |  |  |  | | | | | | | |
| Intrapartum Antibiotic (IPA) Exposure (ref: no IPA) | 1.03 | | 0.84, 1.26 | 0.811 |  |  |  |  |  |  |  |  |
| Sanitation and Hygiene |  | |  |  |  |  |  |  |  |  |  |  |
| Type of toilet (ref: flush toilet)  Squat toilet/Pit latrine  Communal/Others | 0.93  1.04 | | 0.70, 1.23  0.77, 1.39 | 0.613  0.798 |  |  |  |  |  |  |  |  |
| Access to Running Water (ref: at least 4 days in a week)  Irregular or 2-3 days/week  Less than once/week | 1.03  1.15 | | 0.79, 0.83  0.96, 1.39 | 0.790  0.126 |  |  |  |  |  |  |  |  |

Newborns with LBW were more likely to develop GNB sepsis that are resistant to nearly one additional class of antibiotics, compared to those with normal birthweight (increase in MDRscore=0.95 points; 95% CI=0.31, 1.59) (**Table 3**). LOS and birthweight were the only variables included in the multivariable analysis. With birthweight held constant, the MDR score for newborns who developed LOS was higher by 0.59 points (95% CI= 0.02,1.16) compared to newborns with EOS.

**Table 3**: Associations between neonatal, maternal and environmental factors and multidrug resistance (MDR score of 0-9) among 119 newborns with gram-negative sepsis

|  | Univariable Analysis | | | | Multivariable Model | | | | | |
| --- | --- | --- | --- | --- | --- | --- | --- | --- | --- | --- |
| Variable | **β-coeffic-ient** | | **95% CI** | **p-value** | **Variable** | **β-coeffi-cient** | | **95% CI** | **p-value** | |
| Newborns’ Characteristics |  |  | |  |  | | | | | |
| Female (ref: male) | -0.04 | -0.58, 0.51 | | 0.892 |  |  |  |  |  |  |
| Preterm (binary)  <37 weeks (ref: ≥37 weeks)  Preterm (categorical)  (ref: ≥37 weeks)  Moderate/Late (32-<37 weeks)  Very/Extremely (<32weeks) | 0.53  0.67  0.05 | -0.06, 1.11  0.06, 1.28  -0.80, 0.89 | | 0.076  0.032  0.913 |  |  |  |  |  |  |
| Low Birthweight (binary)  <2500g (ref: normal ≥2500g)  Birthweight (categorical)  (ref: normal ≥2500g)  Low (1500-<2500g)  Very/Extremely Low (<1500g) | 0.95  0.96  0.93 | 0.31, 1.59  0.26, 1.66  0.20, 1.66 | | 0.004  0.007  0.013 | Low birthweight  as binary (<2500g) | | 0.81 | 0.17, 1.45 | | 0.013 |
| Outborn (ref: inborn) | -0.23 | -0.79, 0.33 | | 0.419 |  | | | | | |
| Caesarian delivery (ref: vaginal) | -0.16 | -0.63, 0.30 | | 0.486 |  |  |  |  |  |  |
| Onset of Sepsis |  |  | |  |  | | | | | |
| Late-onset sepsis (ref: early-onset) | 0.60 | 0.02, 1.19 | | 0.043 | Late-onset sepsis | | 0.59 | 0.02, 1.16 | | 0.044 |
| Maternal Antibiotics |  |  | |  |  | | | | | |
| Intrapartum Antibiotic (IPA) Exposure (ref: no IPA) | 0.10 | -0.66, 0.86 | | 0.796 |  |  |  |  |  |  |
| Sanitation and Hygiene |  |  | |  |  |  |  |  |  |  |
| Type of toilet (ref: flush toilet)  Squat toilet/Pit latrine  Communal/Others | -0.02  0.16 | -1.18, 1.14  -1.13, 1.45 | | 0.968  0.807 |  |  |  |  |  |  |
| Access to Running Water (ref: at least 4 days/week)  Irregular or 2-3 days/week  Less than once/week | 0.25  0.21 | -0.46, 0.96  -0.55, 0.98 | | 0.486  0.583 |  |  |  |  |  |  |

**Discussion**

The majority of GNB infections among newborns enrolled in Addis Ababa, Ethiopia were resistant to the first-line antibiotics, ampicillin, and gentamicin, consistent with previous results of combined BARNARDS countries (Sands *et al.*, in press; Thomson *et al.*, in press). This study assessed the extent of AMR against a wide range of antibiotics and identified potential alternatives with sensitive antimicrobial activity against GNB sepsis among newborns. In addition to seeking to improve the health outcomes of newborns diagnosed with GNB sepsis, the high prevalence of MDR in this study demonstrates the importance of addressing the growing threat of AMR in developing countries.

The high rates (>80%) of MDR to ampicillin, gentamicin, and the third-generation cephalosporins to routine antibiotics for sepsis at health facilities are concerning and consistent with recent findings in Ethiopia and across sub-Saharan Africa (sSA)[7, 24]. Resistance to amikacin and carbapenems was low (<1%) in this study. This is in line with findings from a meta-analysis which estimated the resistance of *Klebsiella spp*, the most predominant GNB, to amikacin and carbapenems to be 5% and 0% in East Africa, with 14% and 4% as the average for sSA, respectively[24]. However, the prevalence of resistance to amikacin and carbapenems were much higher in other regions. The resistance of GNB isolates to amikacin in North Africa and the Middle East was up to 88% while their prevalence of carbapenem-resistant GNB was as high as 94%[32, 43, 44]. These suggest regional differences in the distribution patterns of AMR. To the best of our knowledge, there was no prior study published from Ethiopia on the prevalence of piperacillin-tazobactam resistance among GNB isolates, which was 39% in this study. However, a pooled analysis from sSA reported a similar prevalence of piperacillin-tazobactam resistance among *Klebsiella spp.,* at 37%[24]. The high rate of resistance to routinely used drugs, which were once effective could serve as an indication that resistance to antibiotics currently found to be efficacious against GNB may rapidly increase within a population if they become first-line therapy[21, 33]. This is further supported by the moderate rate of resistance to ciprofloxacin, an antibiotic, not usually used in children but frequently prescribed for treating infections among older individuals in Ethiopia[45]. This highlights the urgency for more judicious use of antibiotics across all age groups within a population.

The WHO recommends empirical treatment with antibiotics, based on clinical signs when neonatal sepsis is suspected, before laboratory confirmation of diagnosis[17] because of the rapid rate of disease progression and the limited access to laboratory evaluations in sSA countries that have the highest burden of neonatal sepsis[19]. The ideal empirical antibiotic would be inexpensive and would cover common GPB and GNB without increasing AMR[19]. As current evidence suggests the need for new treatment guidelines on antibiotics for neonatal sepsis, it may be necessary to consider regional differences in antibiotic sensitivity before choosing new empirical antibiotics however, globalization may aid the transmission of antibiotic-resistant strains of GNB to areas where an antibiotic had not been introduced[46, 47]. Based on its high efficacy against GNB in this study and others, amikacin could be a potential drug to consider in Ethiopia and across sSA. Amikacin has also proven to be much more effective than gentamicin when used for treating neonatal sepsis in other countries, although they both share similar limitations of administration via intravenous route and the potential to harm the kidneys[19, 48]. Another alternative is piperacillin-tazobactam, which has a fair coverage of GNB, in Ethiopia and sSA, and has been associated with better treatment outcomes in practice than ampicillin or gentamicin[24, 49]. Despite demonstrating a good coverage of GNB sepsis in this study, the increasing global resistance and cost of carbapenems would prevent them from being recommended for widespread use, however, they may be feasible options for use as second-line therapy[50]. Tigecycline, fosfomycin and levofloxacin also demonstrated high efficacy against GNB in this study and have been used as salvage therapies for extensively drug-resistant infections in newborns. However, levofloxacin[51] is not recommended for routine use because of its potential adverse effect on the musculoskeletal system of children while data on the safety, tolerability and dosing of tigecycline[52] and fosfomycin [53]in newborns are limited. Additional research is needed on the safety, pharmacokinetics and cost-effectiveness, of prospective sensitive antibiotics, among newborns, especially those born preterm[54, 55]. In settings without microbiology capacity, treating newborns with clinical symptoms of sepsis with “big gun” antibiotics may do more harm than good by increasing antibiotic resistance or adverse side effects. Furthermore, the mortality outcome of these sensitive antibiotics should be taken from the experience of other countries utilizing amikacin or piperacillin-tazobactam as a first-line regiment.

Preterm birth and low birthweight (LBW) are well-known risk factors for neonatal sepsis. Hence, the likely explanation for the majority of newborns in our study being LBW, and most of the newborns with LBW in our study were born preterm. Preterm birth and LBW were also found to be significant risk factors for AmpGen resistance and MDR, especially among newborns with early-onset sepsis (EOS). Evidence supporting the increased risk of antibiotic-resistant infections with preterm birth was found in two studies[16, 32]. The less developed immune system among preterm newborns, generally thought to be one of the underlying reasons for increased sepsis among preterm newborns, does not directly explain the higher risk for antibiotic-resistant infections[29]. The majority of preterm newborns in this study had EOS which may be associated with maternal intrapartum transmission of pathogens that colonize the mothers’ gastrointestinal or reproductive tract[28, 30]. Further research would be needed to understand whether this increased risk of AMR is due to unexplained host factors among preterm newborns that select for resistant pathogens, or whether the mothers of preterm newborns and newborns with LBW are more likely to have a higher prevalence of colonization by antibiotic-resistant strains of GNB.

Late-onset sepsis (LOS) was associated with an increased risk of MDR in this study and the risk was not particularly higher among babies with LBW. This suggests that antibiotic-resistant GNB are likely to be more prevalent within the primary sources of LOS in Ethiopia. Unlike EOS, sources of infection in LOS have been attributed to be more from horizontal contamination post-delivery than vertical transmission from the mother during labor[27]. GNB infections in LOS can be community-acquired or hospital-acquired, especially when newborns are admitted in neonatal intensive care units[27]. Prevalence of antibiotic resistance is high among hospital-acquired GNB infections and with poor infection control practices[56]. Unsanitary conditions in the environment have also been reported as risk factors for AMR[57]. In this study, the risk of MDR was increased with unsanitary conditions such as lack of access to running water within households but the association did not reach statistical significance, possibly due to the limited sample size. Future research to understand the distribution of community and hospital-acquired LOS in Ethiopia and identify predominant sources of infections in these settings would help to guide the distribution of resources for interventions towards reducing both LOS and the associated AMR.

However, the clustered occurrence of some *Klebsiella pneumoniae* (*Kp*) strains suggests possible outbreaks and may provide clues about infection sources in this study. Strains ST35 and ST37, which were responsible for more than half of all GNB sepsis in this study, have been associated with MDR and reported to be sources of outbreaks within NICU settings outside sSA[58-60]. Both ST35 and ST37 have also been detected in the feces of healthy adults and animals in the community[61]. The incidence of ST37 was much higher among newborns that were inborn, delivered via Caesarean section and had LOS, strongly suggesting that majority of ST37 infections were hospital-acquired. Community outbreak and vertical transmission seemed more plausible for ST218 cases, which occurred within a month and mostly among newborns that were outborn, delivered vaginally and had EOS. With the extreme case fatality rate of 80% observed with ST218 cases, all having the KL57 serotype, it is more likely that the newborns in this study were infected with the hypervirulent type of ST218/KL57, which has been reported among patients at a tertiary hospital about 350km from Addis Ababa in Jimma, Ethiopia, likewise other countries[62, 63]. While there was no difference in the incidence of ST35 between inborn and outborn, the higher incidence of ST35 and ST218 among newborns delivered vaginally may necessitate the screening of pregnant women for colonization by these strains when community outbreaks are suspected. Except for ST37, ST711 and ST985, eight of the 11 *Kp* strains identified in this study were also found among the Jimma patients, suggesting that some of these strains may be endemic to the region. However, BARNARDS study in Ethiopia was limited to a single site; hence, no further comparison could be made with the incidence of *Kp* strains in the community or other NICU settings closer to Addis Ababa. There is a need for surveillance on the K*p* strains responsible for the most neonatal infections and mortality within hospitals and community settings across Ethiopia and sSA. It is imperative to reinforce infection control practices in Ethiopian NICU settings to prevent the spread of deadly *Kp* strains especially because of the limited facilities for early detection.

Intrapartum exposure to ampicillin had been linked with ampicillin-resistant type of neonatal sepsis and this was consistent with findings in our study, not only for ampicillin but also for ceftriaxone, the more frequently used intrapartum antibiotic in this study[35]. There was an increased prevalence of ceftriaxone-resistant GNB isolates with intrapartum exposure to ceftriaxone. However, the higher prevalence of ampicillin and ceftriaxone-resistant GNB sepsis did not reach statistical significance, most likely because of the fewer number of individuals exposed to the antibiotics in this study. Exposure to intrapartum antibiotics was not found to be a significant risk factor for drug resistance to other antibiotics not used as intrapartum antibiotics in this study, consistent with prior studies[37]. Considering that one in four newborns with GNB sepsis received intrapartum antibiotics, it may be necessary to assess whether the supposed benefit of recommending intrapartum antibiotics outweighs the burden from increased antibiotic-resistant GNB infections among newborns and the rationale for intrapartum antibiotics especially in settings where the there is no evidence for Group B streptococcus early-onset sepsis[39].

A limitation of this study is that we were not able to characterize all GNB isolates during the study due to loss of viability hence; the numbers of GNB total did not match the total WGS. In addition, antimicrobial sensitivity testing was conducted separately for constituents of antibiotic combinations, however, a synergistic effect is unlikely *in vivo* when resistance has been demonstrated to the individual components of a combined therapy *in vitro*[64]. For instance, the high mortality rates observed among newborns treated with AmpGen for AmpGen-resistant GNB sepsis (independent resistance to ampicillin and gentamicin *in vitro*) were similar to those among newborns who received no antibiotics for their GNB infections in this study. Evidence from this study confirmed that newborns are more likely to survive GNB infections if treated with the appropriate antibiotics. The high case-fatality among newborns with GNB sepsis may be related to ineffective first-line antibiotics.

**Conclusion**

The current study revealed a high prevalence of resistance to first-line therapy for neonatal sepsis among GNB isolates in Ethiopia. We found lower resistance to amikacin, piperacillin-tazobactam, and carbapenems. There is a need to reassess the current first-line treatment options with antibiotics that have shown sensitivity against GNB, after they have been evaluated for safety, feasibility and availability. Stronger microbiology laboratory capacity to diagnose AMR is required in countries with high burden of neonatal sepsis and high case fatality to make clinical and policy decisions. Preterm birth, LBW, and LOS were identified as risk factors for MDR, and these could provide directions for possible interventions directed towards mitigating AMR in neonatal infections. Improving infection control practices, antimicrobial stewardship regarding intrapartum antibiotics and exploring recent alternative therapeutic options to antimicrobials may reduce the prevalence of antibiotic-resistant GNB sepsis among newborns.

**Contributors**

GJC and DB designed and led the BARNARDS-Ethiopia study. SS, MA, GM contributed to the proposal development. OA, SS and GJC conceptualized the idea for this manuscript. SS and OAO supervised the microbiology wing while MA and GM supervised the clinical wing of the study. OA, FG, KVW, GJC drafted the analysis plan. KVW managed, maintained, and validated the research data. SS, OAO, RA, ZG, MA and GM managed the samples from patients and interpreted the laboratory and clinical findings. SS and KS coordinated the microbiological analysis. MJC, KT, KS coordinated the MIC, identified isolates, and conducted the genomic analysis with TRW. RM contributed to study management and data reconciliation. OA conducted the data analysis, which was audited by KVW. OA drafted the first manuscript and created the tables and figures. All authors contributed intellectually to the preparation of the final draft of the manuscript, reviewed and approved the manuscript before submission.

**Acknowledgments**

We thank the mothers and newborns who participated in BARNARDS-Ethiopia study. We acknowledge the BARNARDS-Ethiopia team Redeat Workneh, Tefere Biteye, Yahya Mohammed and supervisors and data collectors. We appreciate our colleagues at St. Paul’s Hospital Millennium Medical College, Boston Children’s Hospital and Harvard T. H. Chan School of Public Health for their unreserved support during the undertaking of this work, especially Drs. Wendemaggen Gezahegn and Balkachew Nigatu. We would like to acknowledge the clinicians and researchers that provided advice and guidance during BARNARDS and the BARNARDS network (<https://barnards-group.com>). The laboratory work has been coordinated and completed with Cardiff University and we would like to acknowledge Ana Ferreira, Edward Portal, Calie Dyer and Jordan Mathias.

**References**

1.	Newborns: reducing mortality [Internet] [updated 19 September 2020; cited 2021 February 2]. Available from: <https://www.who.int/news-room/fact-sheets/detail/newborns-reducing-mortality>.

2.	Garces AL, McClure EM, Pérez W, Hambidge KM, Krebs NF, Figueroa L, et al. The Global Network Neonatal Cause of Death algorithm for low‐resource settings. Acta Paediatrica. 2017;106(6):904-11.

3.	Fottrell E, Osrin D, Alcock G, Azad K, Bapat U, Beard J, et al. Cause-specific neonatal mortality: analysis of 3772 neonatal deaths in Nepal, Bangladesh, Malawi and India. Archives of Disease in Childhood-Fetal and Neonatal Edition. 2015;100(5):F439-F47.

4.	Neonatal mortality data [Internet] [updated September 2020; cited 2021 Feburary 2]. Available from: <https://data.unicef.org/resources/dataset/neonatal-mortality-data/>.

5.	Ethiopia- UNICEF Data [Internet] [cited 2021 February 2]. Available from: <https://data.unicef.org/wp-content/uploads/country_profiles/Ethiopia/country%20profile_ETH.pdf>.

6.	Moges F, Eshetie S, Yeshitela B, Abate E. Bacterial etiologic agents causing neonatal sepsis and associated risk factors in Gondar, Northwest Ethiopia. BMC pediatrics. 2017;17(1):1-10.

7.	Sorsa A, Früh J, Stötter L, Abdissa S. Blood culture result profile and antimicrobial resistance pattern: a report from neonatal intensive care unit (NICU), Asella teaching and referral hospital, Asella, south East Ethiopia. Antimicrobial Resistance & Infection Control. 2019;8(1):1-6.

8.	Sorsa A. Epidemiology of neonatal sepsis and associated factors implicated: observational study at neonatal intensive care unit of Arsi University Teaching and Referral Hospital, South East Ethiopia. Ethiopian journal of health sciences. 2019;29(3).

9.	Negussie A, Mulugeta G, Bedru A, Ali I, Shimeles D, Lema T, et al. Bacteriological profile and antimicrobial susceptibility pattern of blood culture isolates among septicemia suspected children in selected hospitals Addis Ababa, Ethiopia. International journal of biological and medical research. 2015;6(1):4709.

10.	Dagnew M, Yismaw G, Gizachew M, Gadisa A, Abebe T, Tadesse T, et al. Bacterial profile and antimicrobial susceptibility pattern in septicemia suspected patients attending Gondar University Hospital, Northwest Ethiopia. BMC research notes. 2013;6(1):1-7.

11.	Exner M, Bhattacharya S, Christiansen B, Gebel J, Goroncy-Bermes P, Hartemann P, et al. Antibiotic resistance: What is so special about multidrug-resistant Gram-negative bacteria? GMS hygiene and infection control. 2017;12.

12.	Vaidya VK. Horizontal transfer of antimicrobial resistance by extended-spectrum β lactamase-producing enterobacteriaceae. Journal of laboratory physicians. 2011;3(1):37.

13.	Liang LD, Kotadia N, English L, Kissoon N, Ansermino JM, Kabakyenga J, et al. Predictors of mortality in neonates and infants hospitalized with sepsis or serious infections in developing countries: a systematic review. Frontiers in pediatrics. 2018;6:277.

14.	Dramowski A, Madide A, Bekker A. Neonatal nosocomial bloodstream infections at a referral hospital in a middle-income country: burden, pathogens, antimicrobial resistance and mortality. Paediatrics and international child health. 2015;35(3):265-72.

15.	Blomberg B, Manji KP, Urassa WK, Tamim BS, Mwakagile DS, Jureen R, et al. Antimicrobial resistance predicts death in Tanzanian children with bloodstream infections: a prospective cohort study. BMC infectious diseases. 2007;7(1):1-14.

16.	Hyde TB, Hilger TM, Reingold A, Farley MM, O’Brien KL, Schuchat A. Trends in incidence and antimicrobial resistance of early-onset sepsis: population-based surveillance in San Francisco and Atlanta. Pediatrics. 2002;110(4):690-5.

17.	WHO. Pocket book of hospital care for children: guidelines for the management of common childhood illnesses: World Health Organization; 2013.

18.	Woldu MA, Guta MB, Lenjisa JL, Tegegne GT, Tesafye G, Dinsa H. Assessment of the incidence of neonatal sepsis, its risk factors, antimicrobials use and clinical outcomes in Bishoftu General Hospital, neonatal intensive care unit, Debrezeit-Ethiopia. Int J Contemp Pediatrics. 2017;1(3):135-41.

19.	Fuchs A, Bielicki J, Mathur S, Sharland M, Van Den Anker J. Antibiotic use for sepsis in neonates and children: 2016 evidence update. WHO Reviews. 2016.

20.	Laxminarayan R, Matsoso P, Pant S, Brower C, Røttingen J-A, Klugman K, et al. Access to effective antimicrobials: a worldwide challenge. The Lancet. 2016;387(10014):168-75.

21.	Enweronu-Laryea C, Newman M. Changing pattern of bacterial isolates and antimicrobial susceptbility in neonatal infections in Korle Bu Teaching Hospital, Ghana. East African medical journal. 2007;84(3):136-40.

22.	Kabwe M, Tembo J, Chilukutu L, Chilufya M, Ngulube F, Lukwesa C, et al. Etiology, antibiotic resistance and risk factors for neonatal sepsis in a large referral center in Zambia. The Pediatric infectious disease journal. 2016;35(7):e191-e8.

23.	Crichton H, O’Connell N, Rabie H, Whitelaw A, Dramowski A. Neonatal and paediatric bloodstream infections: Pathogens, antimicrobial resistance patterns and prescribing practice at Khayelitsha District Hospital, Cape Town, South Africa. South African Medical Journal. 2018;108(2):99-104.

24.	Okomo U, Akpalu EN, Le Doare K, Roca A, Cousens S, Jarde A, et al. Aetiology of invasive bacterial infection and antimicrobial resistance in neonates in sub-Saharan Africa: a systematic review and meta-analysis in line with the STROBE-NI reporting guidelines. The Lancet Infectious diseases. 2019;19(11):1219-34.

25.	Yismaw G, Abay S, Asrat D, Yifru S, Kassu A. Bacteriological profile and resistant pattern of clinical isolates from pediatric patients, Gondar University Teaching Hospital, Gondar, Northwest Ethiopia. Ethiopian medical journal. 2010;48(4):293-300.

26.	Magiorakos A-P, Srinivasan A, Carey Rt, Carmeli Y, Falagas Mt, Giske Ct, et al. Multidrug-resistant, extensively drug-resistant and pandrug-resistant bacteria: an international expert proposal for interim standard definitions for acquired resistance. Clinical microbiology and infection. 2012;18(3):268-81.

27.	Camacho-Gonzalez A, Spearman PW, Stoll BJ. Neonatal infectious diseases: evaluation of neonatal sepsis. Pediatric Clinics of North America. 2013;60(2):367.

28.	Chan GJ, Lee AC, Baqui AH, Tan J, Black RE. Risk of early-onset neonatal infection with maternal infection or colonization: a global systematic review and meta-analysis. PLoS Med. 2013;10(8):e1001502.

29.	Collins A, Weitkamp J-H, Wynn JL. Why are preterm newborns at increased risk of infection? Archives of Disease in Childhood-Fetal and Neonatal Edition. 2018;103(4):F391-F4.

30.	Simonsen KA, Anderson-Berry AL, Delair SF, Davies HD. Early-onset neonatal sepsis. Clinical microbiology reviews. 2014;27(1):21-47.

31.	Burroughs T, Najafi M, Lemon SM, Knobler SL. The resistance phenomenon in microbes and infectious disease vectors: implications for human health and strategies for containment: workshop summary. 2003.

32.	Lipsitch M, Samore MH. Antimicrobial use and antimicrobial resistance: a population perspective. Emerging infectious diseases. 2002;8(4):347.

33.	Yusef D, Shalakhti T, Awad S, Algharaibeh Ha, Khasawneh W. Clinical characteristics and epidemiology of sepsis in the neonatal intensive care unit in the era of multi-drug resistant organisms: a retrospective review. Pediatrics & Neonatology. 2018;59(1):35-41.

34.	Ashkenazi-Hoffnung L, Melamed N, Ben-Haroush A, Livni G, Amir J, Bilavsky E. The association of intrapartum antibiotic exposure with the incidence and antibiotic resistance of infantile late-onset serious bacterial infections. Clinical pediatrics. 2011;50(9):827-33.

35.	Bromiker R, Ernest N, Meir MB, Kaplan M, Hammerman C, Schimmel MS, et al. Correlation of bacterial type and antibiotic sensitivity with maternal antibiotic exposure in early-onset neonatal sepsis. Neonatology. 2013;103(1):48-53.

36.	Glasgow TS, Young PC, Wallin J, Kwok C, Stoddard G, Firth S, et al. Association of intrapartum antibiotic exposure and late-onset serious bacterial infections in infants. Pediatrics. 2005;116(3):696-702.

37.	Mercer BM, Carr TL, Beazley DD, Crouse DT, Sibai BM. Antibiotic use in pregnancy and drug-resistant infant sepsis. American journal of obstetrics and gynecology. 1999;181(4):816-21.

38.	Schrag SJ, Hadler JL, Arnold KE, Martell-Cleary P, Reingold A, Schuchat A. Risk factors for invasive, early-onset Escherichia coli infections in the era of widespread intrapartum antibiotic use. Pediatrics. 2006;118(2):570-6.

39.	WHO. World Health Organization recommendation on intrapartum antibiotic administration to women with group B Streptococcus (GBS) colonization for prevention of early neonatal GBS infection. WHO Reprod Heal Libr. 2015.

40.	Flidel-Rimon O, Friedman S, Leibovitz E, Shinwell ES. The use of piperacillin/tazobactam (in association with amikacin) in neonatal sepsis: efficacy and safety data. Scandinavian journal of infectious diseases. 2006;38(1):36-42.

41.	Breakpoint tables for interpretation of MICs and zone diameters, version 10.0,

2020 [cited 2021 February 2]. Available from: <https://www.eucast.org/fileadmin/src/media/PDFs/EUCAST_files/Breakpoint_tables/v_9.0_Breakpoint_Tables.pdf>.

42.	Bursac Z, Gauss CH, Williams DK, Hosmer DW. Purposeful selection of variables in logistic regression. Source code for biology and medicine. 2008;3(1):1-8.

43.	Almohammady MN, Eltahlawy EM, Reda NM. Pattern of bacterial profile and antibiotic susceptibility among neonatal sepsis cases at Cairo University Children Hospital. Journal of Taibah University Medical Sciences. 2020;15(1):39-47.

44.	Mohsen L, Ramy N, Saied D, Akmal D, Salama N, Haleim MMA, et al. Emerging antimicrobial resistance in early and late-onset neonatal sepsis. Antimicrobial Resistance & Infection Control. 2017;6(1):1-9.

45.	Worku F, Tewahido D. Retrospective assessment of antibiotics prescribing at public primary healthcare facilities in Addis Ababa, Ethiopia. Interdisciplinary perspectives on infectious diseases. 2018;2018.

46.	Munita JM, Arias CA. Mechanisms of antibiotic resistance. Virulence mechanisms of bacterial pathogens. 2016:481-511.

47.	Senok AC, Botta GA, Soge OO. Emergence and spread of antimicrobial-resistant pathogens in an era of globalization. Hindawi; 2012.

48.	WHO. Second meeting of the subcommittee of the expert committee on the selection and use of essential medicines. Geneva: World Health Organization. 2018.

49.	Chong E, Reynolds J, Shaw J, Forur L, Delmore P, Uner H, et al. Results of a two-center, before and after study of piperacillin–tazobactam versus ampicillin and gentamicin as empiric therapy for suspected sepsis at birth in neonates⩽ 1500 g. Journal of Perinatology. 2013;33(7):529-32.

50.	Meletis G. Carbapenem resistance: overview of the problem and future perspectives. Therapeutic advances in infectious disease. 2016;3(1):15-21.

51.	Goldman JA, Kearns G. Fluoroquinolone use in paediatrics: focus on safety and place in therapy. World Health Organization, Geneva, Switzerland. 2011:1-13.

52.	İpek MS, Gunel ME, Ozbek E. Tigecycline Use in Neonates: 5-Year Experience of a Tertiary Center. Journal of Pediatric Infectious Diseases. 2019;14(03):103-7.

53.	Williams PC. Potential of fosfomycin in treating multidrug‐resistant infections in children. Journal of paediatrics and child health. 2020;56(6):864-72.

54.	Raymond B. Five rules for resistance management in the antibiotic apocalypse, a road map for integrated microbial management. Evolutionary applications. 2019;12(6):1079-91.

55.	Pokhrel B, Koirala T, Shah G, Joshi S, Baral P. Bacteriological profile and antibiotic susceptibility of neonatal sepsis in neonatal intensive care unit of a tertiary hospital in Nepal. BMC pediatrics. 2018;18(1):1-8.

56.	Peters L, Olson L, Khu DT, Linnros S, Le NK, Hanberger H, et al. Multiple antibiotic resistance as a risk factor for mortality and prolonged hospital stay: A cohort study among neonatal intensive care patients with hospital-acquired infections caused by gram-negative bacteria in Vietnam. PloS one. 2019;14(5):e0215666.

57.	Fletcher S. Understanding the contribution of environmental factors in the spread of antimicrobial resistance. Environmental health and preventive medicine. 2015;20(4):243-52.

58.	Frenk S, Rakovitsky N, Temkin E, Schechner V, Cohen R, Kloyzner BS, et al. Investigation of Outbreaks of Extended-Spectrum Beta-Lactamase-Producing Klebsiella Pneumoniae in Three Neonatal Intensive Care Units Using Whole Genome Sequencing. Antibiotics. 2020;9(10):705.

59.	Yan J, Wang M, Zheng P, Tsai L, Wu J. Associations of the major international high-risk resistant clones and virulent clones with specific ompK36 allele groups in Klebsiella pneumoniae in Taiwan. New microbes and new infections. 2015;5:1-4.

60.	Chen D, Hu X, Chen F, Li H, Wang D, Li X, et al. Co-outbreak of ST37 and a novel ST3006 Klebsiella pneumoniae from multi-site infection in a neonatal intensive care unit: a retrospective study. bioRxiv. 2018:334169.

61.	Zhong X-s, Li Y-z, Ge J, Xiao G, Mo Y, Wen Y-q, et al. Comparisons of microbiological characteristics and antibiotic resistance of Klebsiella pneumoniae isolates from urban rodents, shrews, and healthy people. BMC microbiology. 2020;20(1):1-8.

62.	Sewunet T, Asrat D, Woldeamanuel Y, Ny S, Westerlund F, Aseffa A, et al. High prevalence of bla CTX-M-15 and nosocomial transmission of hypervirulent epidemic clones of Klebsiella pneumoniae at a tertiary hospital in Ethiopia. JAC-Antimicrobial Resistance. 2021;3(1):dlab001.

63.	Liu C, Du P, Xiao N, Ji F, Russo TA, Guo J. Hypervirulent Klebsiella pneumoniae is emerging as an increasingly prevalent K. pneumoniae pathotype responsible for nosocomial and healthcare-associated infections in Beijing, China. Virulence. 2020;11(1):1215-24.

64.	Mahajan G, Thomas B, Parab R, Patel ZE, Kuldharan S, Yemparala V, et al. In vitro and in vivo activities of antibiotic PM181104. Antimicrobial agents and chemotherapy. 2013;57(11):5315-9.
